# Toward an Objective Assessment of Implementation Processes for Innovations in Health Care: Psychometric Evaluation of the Normalization Measure Development (NoMAD) Questionnaire Among Mental Health Care Professionals

**DOI:** 10.2196/12376

**Published:** 2019-02-20

**Authors:** Christiaan Vis, Jeroen Ruwaard, Tracy Finch, Tim Rapley, Derek de Beurs, Henk van Stel, Britt van Lettow, Mayke Mol, Annet Kleiboer, Heleen Riper, Jan Smit

**Affiliations:** 1 Department of Clinical, Neuro-, & Developmental Psychology Faculty of Behavioural and Movement Sciences Vrije Universiteit Amsterdam Amsterdam Netherlands; 2 Mental Health Amsterdam Public Health Research Institute Amsterdam Netherlands; 3 Amsterdam UMC, Vrije Universiteit, Psychiatry Amsterdam Public Health Research Institute Amsterdam Netherlands; 4 Research and Innovation GGZ inGeest Specialized Mental Healthcare Amsterdam Netherlands; 5 Department of Nursing, Midwifery & Health Northumbria University Northumbria United Kingdom; 6 Department of Social Work, Education & Community Wellbeing Northumbria University Northumbria United Kingdom; 7 Mental Health Netherlands Institute For Health Services Research (NIVEL) Utrecht Netherlands; 8 Julius Center Research Program Methodology Department of Public Health, Healthcare Innovation & Evaluation and Medical Humanities University Medical Center Utrecht Utrecht Netherlands; 9 Nictiz The Hague Netherlands

**Keywords:** implementation science, eHealth, psychometrics, eMental health, normalization process theory, implementation assessment

## Abstract

**Background:**

Successfully implementing eMental health (eMH) interventions in routine mental health care constitutes a major challenge. Reliable instruments to assess implementation progress are essential. The Normalization MeAsure Development (NoMAD) study developed a brief self-report questionnaire that could be helpful in measuring implementation progress. Based on the Normalization Process Theory, this instrument focuses on 4 generative mechanisms involved in implementation processes: coherence, cognitive participation, collective action, and reflexive monitoring.

**Objective:**

The aim of this study was to translate the NoMAD questionnaire to Dutch and to confirm the factor structure in Dutch mental health care settings.

**Methods:**

Dutch mental health care professionals involved in eMH implementation were invited to complete the translated NoMAD questionnaire. Confirmatory factor analysis (CFA) was conducted to verify interpretability of scale scores for 3 models: (1) the theoretical 4-factor structure, (2) a unidimensional model, and (3) a hierarchical model. Potential improvements were explored, and correlated scale scores with 3 control questions were used to assess convergent validity.

**Results:**

A total of 262 professionals from mental health care settings in the Netherlands completed the questionnaire (female: 81.7%; mean age: 45 [SD=11]). The internal consistency of the 20-item questionnaire was acceptable (.62≤alpha≤.85). The theorized 4-factor model fitted the data slightly better in the CFA than the hierarchical model (Comparative Fit Index=0.90, Tucker Lewis Index=0.88, Root Mean Square Error of Approximation=0.10, Standardized Root Mean Square Residual=0.12, χ^2^_2_=22.5, *P*≤.05). However, the difference is small and possibly not outweighing the practical relevance of a total score and subscale scores combined in one hierarchical model. One item was identified as weak (λ_CA.2_=0.10). A moderate-to-strong convergent validity with 3 control questions was found for the Collective Participation scale (.47≤*r*≤.54, *P*≤.05).

**Conclusions:**

NoMAD’s theoretical factor structure was confirmed in Dutch mental health settings to acceptable standards but with room for improvement. The hierarchical model might prove useful in increasing the practical utility of the NoMAD questionnaire by combining a total score with information on the 4 generative mechanisms. Future research should assess the predictive value and responsiveness over time and elucidate the conceptual interpretability of NoMAD in eMH implementation practices.

## Introduction

### Background

More than 2 decades of research has shown that psychotherapy delivered through the internet, also referred to as eMental Health (eMH) interventions, can be an effective way to treat patients with common mental disorders such as depression and anxiety disorder [1]. Several examples of clinics routinely offering innovative and new eMH services exist, such as the Australian MindSpot clinic [2], GGZ InGeest Mindway [3] and Interapy in the Netherlands [4], Internetpsykiatr in Sweden [5], and Internetpsykatrien in Denmark [6,7]. Despite these examples, and although the technical infrastructure seems to be in place, large-scale use of eMH interventions in routine care is still lower than expected [8]. Knowledge on factors hindering or facilitating implementation is maturing [9,10]. However, measuring implementation outcomes reliably remains a challenge [11,12]. We conducted a psychometric validation study of a recently developed theory-informed implementation measurement instrument: the Normalization MeAsure Development (NoMAD) questionnaire.

### Theoretical Underpinning

Various frameworks and theories for understanding implementation processes and evaluating outcomes exist [13,14]. For example, models such as the Knowledge-to-Action model [15] have been specifically designed to describe and guide implementation processes. Determinant frameworks such as the Consolidated Framework for Implementation Research (CFIR) [16] provide taxonomies of barriers and hindering factors to aid the evaluation of implementation outcomes. Similarly, the Reach Effectiveness-Adoption Implementation Maintenance framework [17] summarizes key indicators for implementation success to inform policy and decision making. Classic psychological behavior change theories such as the Theory of Planned Behavior [18] have been used to study the role of attitudes and intentions in the behavior of individuals involved in and affected by implementation processes. Although such theories can be useful in describing behavior change mechanisms and explaining how change in individuals involved in implementation processes occurs, they do not necessarily consider what people actually do when implementing innovations in health care practice but rather focus on beliefs and attitudes. The Normalization Process Theory (NPT) [19,20] aims to fill this void by specifically looking at the process of implementation.

**Figure 1:**
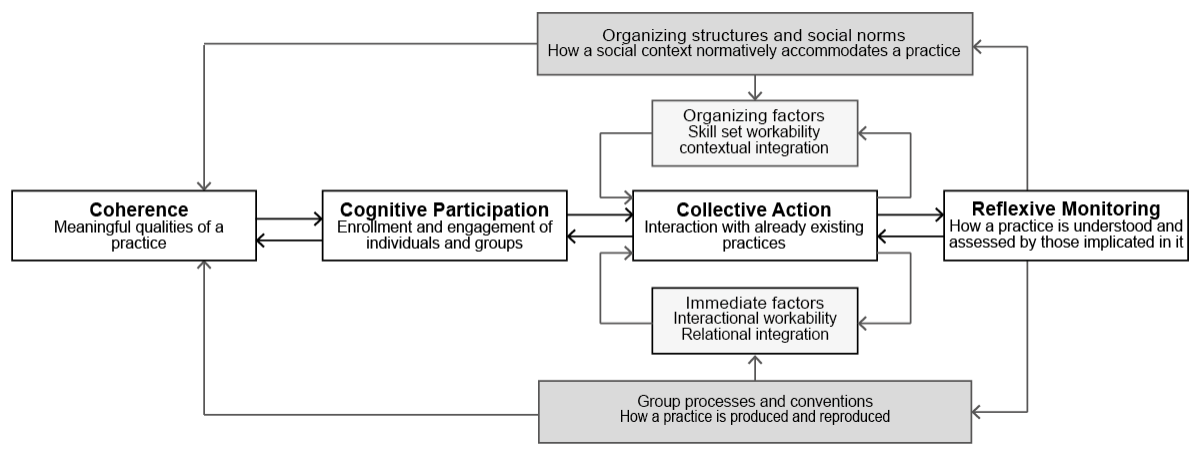
Conceptual model of Normalization Process Theory (NPT): 4 constructs situated in a social and organizational context.

NPT (Figure 1 [19]) states that a normalization process is a process of embedding and integrating health care innovations in routine care as a product of action of individuals and groups. It focuses on the things that people individually and collectively do to normalize an innovation, that is, for it to become part of routine health care practice. NPT is a heuristic tool to understand the work of implementation, embedding, and integration of new practice and the contribution and roles of individuals and groups to this work. According to the theory, 4 mechanistic constructs play a central role in generating the work of implementation:

- Coherence (CO) of the innovation with the goals of daily routine. Individuals and groups go through a process of sense-making to establish the meaningfulness of the innovation for normal service delivery goals and practices.
- Cognitive participation (CP) as a process of enrollment and engagement of individual participants and groups involved in the implementation processes, through which they become committed to the normalization of the innovation.
- Collective action (CA) by individuals and groups to apply the innovation in daily routine. Here, applying an innovation has certain implications as to what and how normalization should be achieved, which requires investments of effort by the participants.
- Reflexive monitoring (RM) through which participants in the implementation process evaluate and appraise the use of the innovation in practice.

These 4 constructs are influenced by group processes and social conventions as well as the organizational factors and social structures people operate in. In turn, this social and organizational context defines factors that promote or inhibit the work of individuals and collectives in implementing innovations in daily routines.

Earlier work showed that NPT has good face validity in designing and evaluating implementation processes of innovations [21]. A recent literature review of 108 studies indicated that NPT successfully aids in the conceptual understanding of implementation processes and outcomes across a wide variety of health care settings [22].

In alignment with the general approach of NPT, the NoMAD study developed a brief self-report questionnaire for the purpose of determining factors likely to affect normalization processes [23-25]. Ultimately, the questionnaire aims to enable (1) assessment of progress toward normalization over time in an implementation project and (2) comparison of normalization (progress or outcomes) between sites in multicenter studies. The NoMAD is intended to be used by people involved in the implementation of innovations in a health care setting and aims to be neutral to the implementation object. The target populations of the instrument are the deliverers and facilitators of the innovation being implemented, such as medical specialists, general practitioners, therapists, nurses, administrators, and managers.

A pool of 46 construct items was generated, appraised, and validated in 5 UK and 1 Australian samples of health care staff (n_pooled_=413) involved in 6 different implementation projects [23-25]. A psychometric evaluation of the initial item pool resulted in a 20-item questionnaire of which the theoretical model approximated the data acceptably and appeared to have good internal consistency (total Normalization Process Scale (NPS): alpha=.89, CO: alpha=.71, CA: alpha=.78, CP: alpha=.81, RM: alpha=.65) [25].

### Objective

We translated the questionnaire into Dutch and aimed to confirm the theoretical factor structure in mental health professionals working to implement eMH in Dutch mental health care settings. We tested 3 factor structures: (1) A 4-factor model to confirm the theorized model, summarizing item scores per construct; (2) A unidimensional model to test whether the items in the questionnaire can be summarized by 1 single factor score; and (3) A hierarchical model to test whether the 4 first-order factors can be expressed in 1 second-order factor. Where the first model aims to capture a more detailed view on implementation processes, the second model might lend support for practical comparison of those processes. The third model might provide a more detailed understanding of normalization processes on the construct level combined with the practical value of the overall total normalization score in 1 measurement model. Conforming to the English validation study [23-25], we explored potential improvements and the questionnaire’s convergent validity with 3 control questions.

## Methods

### Sample and Recruitment

Using a cross-sectional design, mental health professionals with various occupational backgrounds involved in implementing eMH in Dutch routine mental health care practices were invited to complete the NoMAD questionnaire. We defined involvement in implementation as the situation in which respondents were in the early stages of using eMH in their occupational tasks. By this, novelty to the respondent in applying such interventions in routine care was assumed. Following the English NoMAD study, an open sampling strategy was applied to obtain a sample of 300 respondents. Considering the commonly applied rule of thumb of 7 to 10 complete cases per item with a minimum of 100 complete cases, we expected this target sample size to provide satisfactory statistical power and precision for estimating the model’s parameters [26,27]. Recruitment targeted mental health professionals involved in using novel eMH interventions in (1) primary care for patients with mild symptomatology (general practitioners or general practice–based mental health nurse specialists), (2) basic care for patients with moderate symptomatology, and (3) specialized care provided by specialists to patients with severe mental health complaints. A total of 3 groups of Dutch mental health professionals were identified as suitable for recruitment:

- Group 1: mental health care professionals in 4 large regional mental health organizations for common mental disorders and post-traumatic stress disorders.
- Group 2: general practice–based mental health nurse specialists, in the context of the national electronic health (eHealth) Monitor survey conducted in 2016 for which panels and profession associations were sampled [28].
- Group 3: attendees at the annual cognitive behavioral therapy (CBT) congress held in the Netherlands in 2016, which attracted a nationwide audience of mental health professionals.

A total of 3 different recruitment strategies were applied. Sample 1 was obtained through convenience sampling by which participants were recruited through key contact persons in various mental health organizations. Sample 2 was obtained through existing respondent panels and professional associations in the context of the national eHealth survey. Participants for samples 1 and 2 were invited by email providing general information about the study, a link to more in-depth information, and an anonymous link to the Web-based survey. Sample 3 was recruited through an information kiosk and leaflets at the annual CBT conference.

### Translation

The classical Brislin approach to questionnaire translation [29] was used to translate the English NoMAD questionnaire into Dutch. A small (N=3) sample of experts in implementing and using eMH interventions were asked to verbalize their thoughts while interpreting the translated items in a cognitive group interview [30]. The interview focused on the interpretation of the questions, the response scales, and the identification of terms that needed to be adjusted and/or rephrased. Problematic items were rephrased to form the final version of the Dutch NoMAD instrument. Back translation by a blinded professional translator confirmed equivalence of semantic meaning of the corresponding individual items by the principal investigator (TF) of the English NoMAD. The final Dutch translation of the questionnaire is included in Multimedia Appendix 1.

### Data Collection

The questionnaire was administered via a commercial Web-based survey system (NETQ Internet Surveys 6.5 [31]). The research team tested the survey for sequencing of the items, technical reliability, and data export procedures. Participants were asked to provide consent for using their (anonymized) data in this study. They provided this digitally through the survey platform before they were allowed access to the survey.

### Normalization MeAsure Development Questionnaire

The NoMAD questionnaire in this study consisted of 3 parts: Part A tapping basic demographic information, Part B collecting general normalization ratings about the current use and likelihood of using the intervention in the future, and Part C comprising 20 items measuring the 4 NPT constructs. Users of the questionnaire are required to tailor the implementation object (ie, intervention) to the context of its application. In this study, the terms (the intervention) were replaced with “eMental health.”

Part A: Demographic variables. In line with the English NoMAD, basic demographic variables were included in the first part of the questionnaire, including gender, age, years of working experience, professional job category, and relevant care sector.

Part B: General normalization items. Part B contained 3 questions addressing perceptions of respondents regarding past, current, and future normality of the intervention. The 3 questions were scored on a 1 to 10 Visual Analogue Scale [32]. To increase comparability to the UK study, these 3 items were added to the questionnaire as control questions to assess its convergent validity, that is, the 3 questions are not to be regarded as an integral part of the core of the NoMAD questionnaire [23-25].

Part C: NPT constructs. Part C consisted of the 20 items representing the NPT constructs in 4 subscales with the following allocation: CO: 4 items; CP: 4 items; CA: 7 items; and RM: 5 items. The 20 original items are listed in Textbox 1.

The items were rated on a 5-point Likert scale (1=completely agree to 5=completely disagree), with an additional response option to indicate if a statement was applicable (0=not applicable). Item 2 (CA.2) in the CA scale is negatively formulated; all other items were formulated in a positive sense. Respondents were required to rate all statements.

The Dutch translations are in Multimedia Appendix 1.

### Scoring

Scale scores were calculated by taking the mean of answered items of a scale. A minimum of 2 items within a scale had to be rated to calculate a scale score. Items rated as “not applicable” were excluded from the calculation. The total NPS score was calculated by taking the mean of all answered items for which complete cases were considered to have less than 15% missing data.

### Data Analyses

Descriptive statistics were calculated to summarize the item and scale scores. Internal consistency of the total score and the 4 theoretical constructs were analyzed by calculating the Cronbach alpha [27] for the pooled dataset. The quality of the construct structure was further assessed by applying a confirmatory factor analysis (CFA) using Structural Equation Modelling (SEM). A total of 3 models were evaluated: (1) the theorized 4-factor model, (2) a unidimensional model, and (3) a hierarchical model. All 3 models included the 20 items from Part C of the questionnaire. The items were scored on a 5-point Likert scale resulting in an ordinal ordering of the data. The sum scale score of the items approximates a continuous scale by which we expected the latent constructs to be normally distributed. The CFA was run with the robust Weighted Least Square Means and Variances (WLSMV) estimator using polychoric correlation matrices [26]. Model fit was assessed by estimating the misfit between the observed and implied covariance matrices using the chi-squared test (χ^2^≤3df). This was supplemented with 4 other fit estimators to strengthen the basis for our conclusions: the Standardized Root Mean Square Residual (SRMR≤0.08) as an absolute index of the average discrepancy between the correlations in the implied model and the observed data; the Root Mean Square Error of Approximation (RMSEA≥0.95) providing a population-based goodness-of-fit indication corrected for model complexity; the Comparative Fit Index (CFI≥0.95) providing an index of goodness-of-fit relative to a null model (ie, no covariances between items); and the Tucker Lewis Index (TLI≥0.95) as an index of goodness-of-fit relative to a null model corrected for model complexity [26,33,34]. The 3 models under evaluation are expected to be nested. We applied the scaled chi-square difference test (χ^2^_diff_ test, analysis of variance) to compare the fit of the 3 models [26].

**Normalization MeAsure Development (NoMAD) questionnaire part C items.**

Coherence (CO):

1. CO.1. I can distinguish [the intervention] from usual ways of working.
2. CO.2. Staff in this organization have a shared understanding of the purpose of [the intervention].
3. CO.3. I understand how [the intervention] affects the nature of my own work.
4. CO.4. I can see the potential value of [the intervention] for my work.

Cognitive participation (CP):

1. CP.1. There are key people who drive [the intervention] forward and get others involved.
2. CP.2. I believe that participating in [the intervention] is a legitimate part of my role.
3. CP.3. I’m open to working with colleagues in new ways to use [the intervention].
4. CP.4. I will continue to support [the intervention].

Collective action (CA):

1. CA.1. I can easily integrate [the intervention] into my existing work.
2. CA.2. [the intervention] disrupts working relationships.
3. CA.3. I have confidence in other people’s ability to use [the intervention].
4. CA.4. Work is assigned to those with skills appropriate to [the intervention].
5. CA.5. Sufficient training is provided to enable staff to implement [the intervention].
6. CA.6. Sufficient resources are available to support [the intervention].
7. CA.7. Management adequately support [the intervention].

Reflexive monitoring (RM):

1. RM.1. I am aware of reports about the effects of [the intervention].
2. RM.2. The staff agree that [the intervention] is worthwhile.
3. RM.3. I value the effects [the intervention] has had on my work.
4. RM.4. Feedback about [the intervention] can be used to improve it in the future.
5. RM.5. I can modify how I work with [the intervention].

Potential improvements to the factor structure were explored by identifying low item-factor loadings (λ<0.3) to ensure that items are meaningfully related to the respective factors [26]. Modification indices (modification index [MI], χ^2^_diff_≥3.84) were assessed to identify item-item error covariances that might improve the model fit.

In the absence of a gold standard for the assessment of normalization, we exploratively used the 3 general normalization items (part B) to assess the convergent validity of the theorized model. We assessed the Pearson correlation coefficients for all 4 constructs and general normalization items and applied the following strength indicators for the correlations: 0≤*r*<.3 is weak, .3≤*r*<.5 is moderate, and *r*≥.5 is strong [35]. These quality indicators were applied in all correlation assessments.

Data cleaning and analyses were performed in RStudio [36,35] using the following packages: psych [37], ggplot2 [38], sjPlot [39], lavaan [40], semPlot [41], and semTools [42].

### Ethical Approval and Consent to Participate

Ethical and scientific approval was granted by the Scientific and Ethical Review Board of the Faculty of Behavioural and Movement Sciences at the VU Amsterdam (file number: VCWE-2016-006).

## Results

### Sample

Over a period of 10 months (May 2016 to February 2017), 262 respondents completed the questionnaire. Table 1 provides an overview of the samples and participant characteristics. On a pooled level, participants were middle-aged (*M=* 45, SD=11), female (81.7%), and had over 11 years working experience in their respective fields (52.9%). The response rate for group 2 was 22.8% (125 out of 547) [28]. For sample groups 1 and 3, response rates are not available because of the convenience and open sampling approach. The time required to complete the questionnaire was 7.56 min on average (SD=6.48, n=134, based on questionnaire log files).

### Scale scores

**Figure 2:**
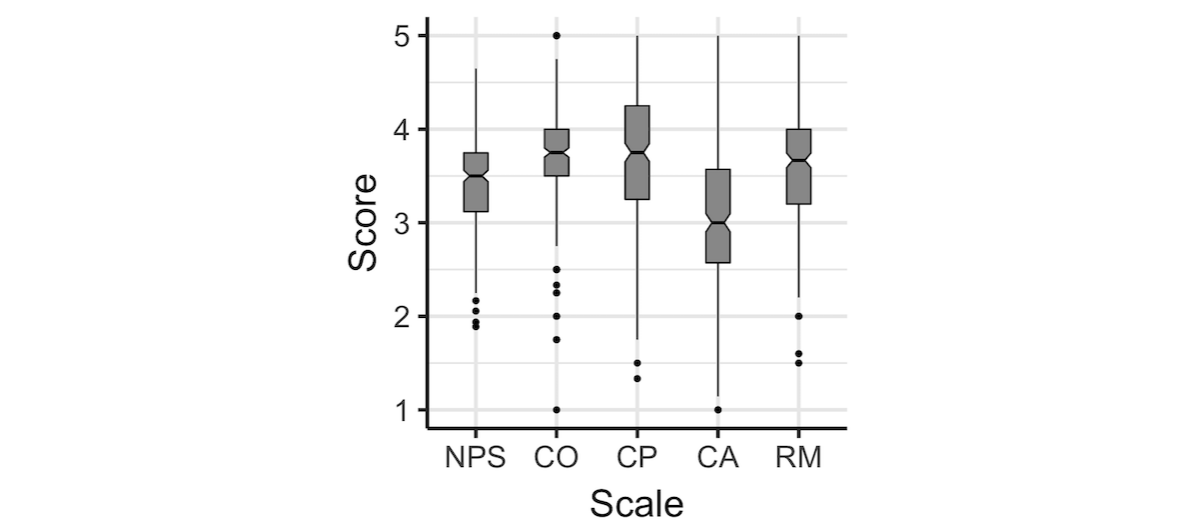
Boxplot of the scale scores for the combined mental health samples. CA: collective action; CO: coherence; CP: cognitive participation; NPS: normalization process scale; RM: reflexive monitoring.

Figure 2 shows the distributional characteristics of the scale scores for the combined samples. The 4 subconstructs (CO, CP, CA, and RM) and the NPS follow similar response patterns. Considering the length of the boxplot for the scales, respondents vary less in responses to items for the CO construct and more for CP and CA. The distributions of 3 subscales appear to have a slight tendency toward agreement with item statements where CA received mostly neutral responses. Most outliers are in the disagreement end of the scales.

**Figure 3:**
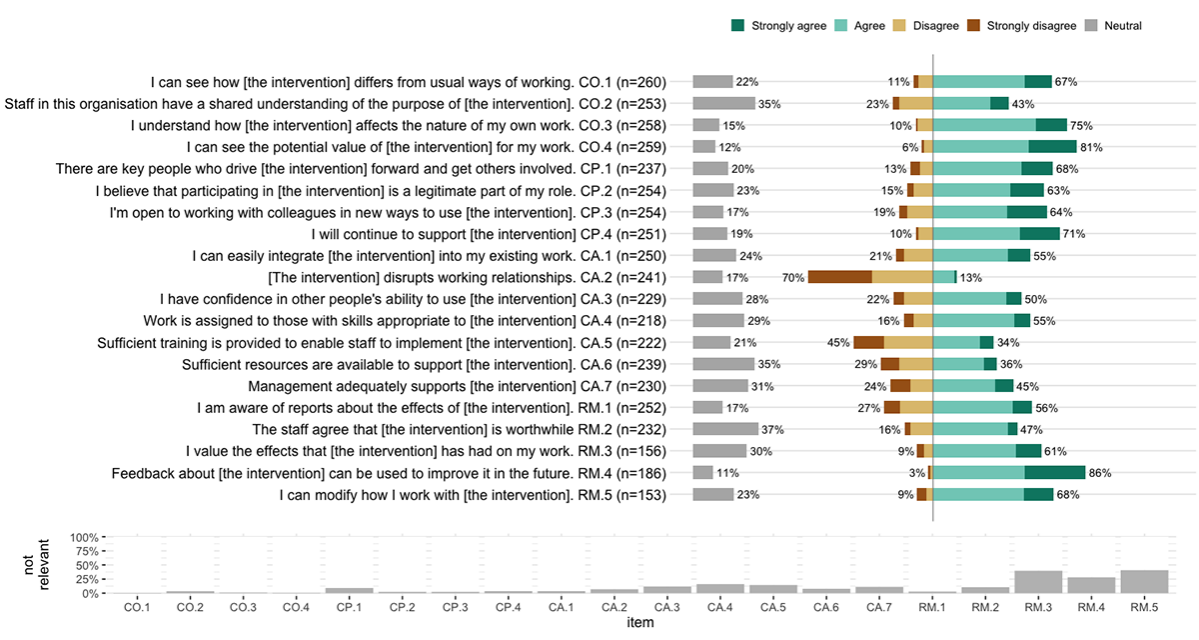
Frequency distribution of item responses. The upper part of the figure shows the percentage of respondents reporting strongly disagree, disagree, agree, or strongly agree. The gray bar coupled to the y-axis indicates the percentage of participants rating an item as “neutral.” The lower part of the figure shows the percentage of respondents who chose to not to rate a specific item (ie, not relevant). CO: coherence, CP: cognitive participation. CA: collective action. RM: reflexive monitoring.

Table 2 shows the mean scale scores, indicating that respondents on average agreed with the item statements. Respondents disagreed considerably with item CA.2, indicating that they did not find the intervention disruptive to working relations (Figure 3).

**Table 1:** Sample composition and demographics of respondents of the Dutch Normalization MeAsure Development questionnaire.

Variable		Pooled	Group 1^a^	Group 2^b^	Group 3^c^
Cases, n (%)		262 (100.0)	115 (43.9)	125 (47.7)	22 (8.4)
Age (years), mean (SD)		46.4 (11)	41.5 (10.7)	48.6 (10.1)	43.1 (11)
**Gender, n (%)**					
	Female	214 (81.7)	91 (79.1)	108 (86.4)	15 (68.2)
**Work experience (years), n (%)**					
	<1	4 (1.7)	3 (1.2)	1 (0.4)	0 (0)
	1-2	16 (6.6)	3 (1.2)	13 (5.4)	0 (0)
	3-5	46 (19.0)	19 (7.9)	27 (11.2)	0 (0)
	6-10	48 (19.8)	21 (8.7)	19 (7.9)	8 (3.3)
	11-15	32 (13.2)	17 (7.0)	13 (5.4)	2 (0.8)
	>15	96 (39.7)	36 (14.9)	52 (21.5)	8 (3.3)
**Sector^d^, n (%)**					
	PC-MH^e^	135 (51.5)	12 (4.6)	122 (46.6)	2 (0.4)
	BC-MH^f^	35 (13.4)	20 (7.6)	8 (3.1)	7 (2.7)
	SC-MH^g^	114 (43.5)	97 (37.0)	0 (0.0)	17 (6.5)

^a^Group 1: mental health care professionals in large regional mental health organizations.

^b^Group 2: general practice–based mental health nurse specialists.

^c^Group 3: mental health professionals attending the annual national cognitive behavioral therapy (CBT) congress.

^d^Sector: respondents could choose multiple answers: primary care-mental health services, basic care-mental health, and specialist care-mental health.

^e^PC-MH: primary care-mental health services.

^f^BC-MH: basic care-mental health.

^g^SC-MH: specialist care-mental health.

**Table 2:** Mean scale scores.

Scale^a^	n^b^	Mean (SD)	Low^c^	High^c^
Normalization process scale (NPS)^c^	221	3.54 (0.51)	2.11	4.85
Coherence (CO)	259	3.70 (0.67)	1.00	5.00
Cognitive participation (CP)	256	3.69 (0.73)	1.33	5.00
Collective action (CA)	227	3.30 (0.69)	1.29	5.00
Reflexive monitoring (RM)	181	3.55 (0.62)	1.50	5.00

^a^For the total NPS scale, a maximum of 15% missingness was allowed. For the sub-scales, a minimum of 2 rated items were needed to calculate a mean.

^b^n varies because of item nonresponse.

^c^Low and High represent the lowest (1) and highest (5) score, respectively, rated by the respondents.

### Internal Consistency

Considering the number of items, the internal consistency of the translated NoMAD questionnaire is good for the total score (alpha_NPS_=.85) and ranges from questionable to acceptable for the subscales (.62≤alpha≤.75; Table 3). Internal consistency improved to good when items were dropped.

### Factor Structure

**Figure 4:**
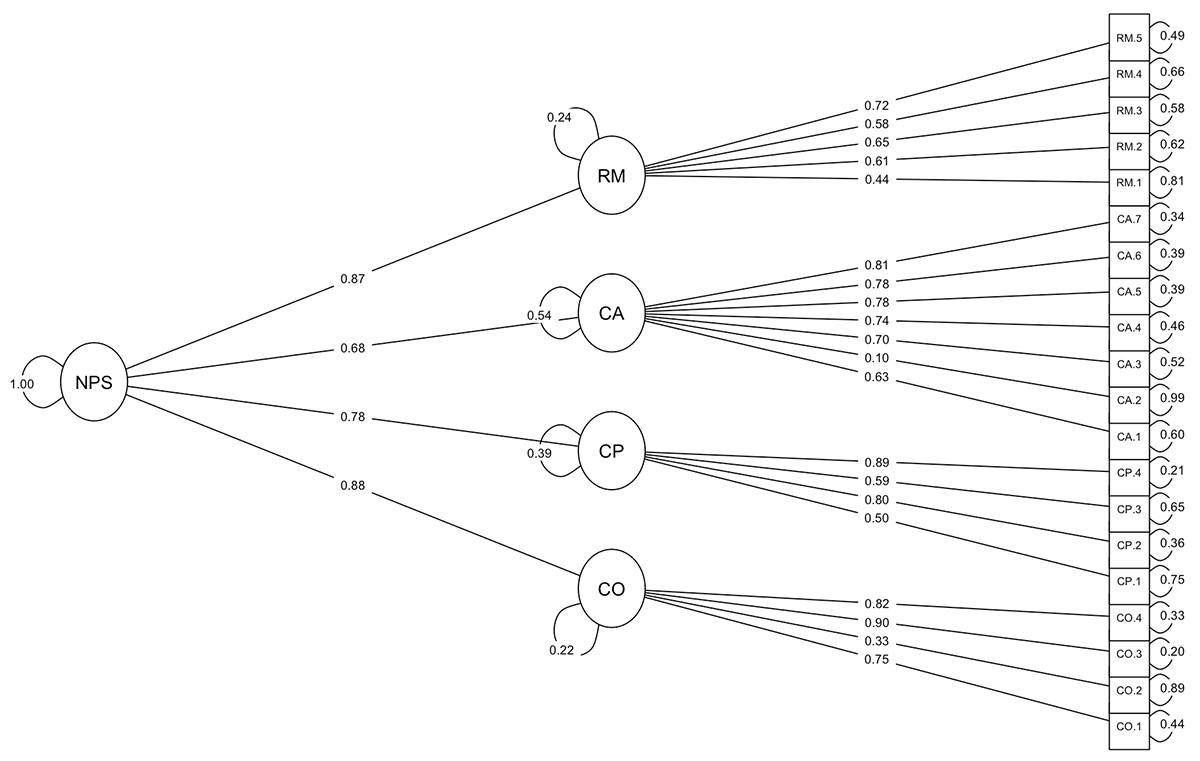
Factor structure of the hierarchical model including item factor loadings and residuals. CA: collective action; CO: coherence; CP: cognitive participation; NPS: normalization process scale; RM: reflexive monitoring.

Table 4 summarizes the CFA results and the fit indices for the 3 models: (1) the first order 4-factor model in which normalization is defined by 4 correlated constructs, (2) the first order unidimensional model, and (3) the hierarchical model in which a second-level factor accounts for the correlations among the 4 first-order factors. Considering the number of items, all 3 models fitted the data reasonably well. Both the 4-factor model and the hierarchical model represented the observed data significantly better than the unidimensional model (respectively: χ^2^_6_=220.7, *P* ≤.05, and χ^2^_4_=198.1, *P* ≤.05). The 4-factor model performed better than the hierarchical model (χ^2^_2_=22.5, *P* ≤.05) with less discrepancy between the obtained and implied data (χ^2^_164_=559.7, SRMR=0.12), better fit per variable (RMSEA=0.10), and better fit relative to a baseline model (CFI=0.90, TLI=0.88). Notwithstanding the significance, the difference for the chi-square test statistic and the fit indices is small and potentially not outweighing the practical relevance of a total summary score and subscale scores combined in one measurement model. Therefore, the factor structure of the hierarchical model is displayed in Figure 4.

**Table 3:** Internal consistency calculated by using Cronbach alpha.

Scale	Cronbach alpha UK^a^	Cronbach alpha NL^b^ (95% CI)	Cronbach alpha, if item dropped	Item-rest correlation
Normalization process scale	.89	.85 (0.82-0.89)	.86 (CA.2^c^)	.03 (CA.2)
Coherence	.71	.71 (0.61-0.81)	.80 (CO.2^d^)	.25 (CO.2)
Cognitive participation	.81	.62 (0.51-0.73)	.75 (CP.1^e^)	.10 (CP.1)
Collective action	.78	.75 (0.69-0.82)	.81 (CA.2)	.00 (CA.2)
Reflexive monitoring	.65	.64 (0.54-0.74)	—^f^	.36 (RM.1^g^)

^a^UK: English validation study results [25].

^b^NL: current Dutch study sample.

^c^CA.2: collective action item 2.

^d^CO.2: coherence item 2.

^e^CP.1: cognitive participation item 1.

^f^No improvement of alpha found.

^g^RM.1: reflexive monitoring item 1.

**Table 4:** Results of the confirmatory factor analysis (CFA). A fourth model is included in the CFA to explore potential improvements only.

Model	n_par_^a^	χ^2b^	df	CFI^c^	TLI^d^	RMSEA^e^	SRMR^f^
Four-factor	106	559.7	164	0.90	0.88	0.10	0.12
Unidimensional	100	837.3	170	0.82	0.80	0.12	0.15
Hierarchical	104	580.9	166	0.89	0.87	0.10	0.12
Hierarchical modified	101	426.1	146	0.93	0.91	0.09	0.11

^a^n_pa__*r*_: number of parameters estimated in the CFA.

^b^χ^2^: scaled chi-squared test.

^c^CFI: Comparative Fit Index.

^d^TLI: Tucker Lewis Index.

^e^RMSEA: Root Mean Square Error of Approximation.

^f^SRMR: Standardized Root Mean Square Residual.

### Potential Model Improvements

We explored possibilities to improve the measurement accuracy and reliability of the proposed hierarchical model. From evaluating the item-factor loadings, it can be concluded that item CA.2 has a weak relationship with CA (λ=0.10), indicating that less than 1% of the variance in this item is explained by this factor. This is confirmed by the “Cronbach alpha-if-item-dropped” statistic indicating an improvement in the measured internal consistency of the theorized model when this item is removed (Table 3). MIs were consulted for the 4-factor model and 2 error term covariances—CA.3 to CA.4 (MI=76.9, δ=0.56) and CP.3 to CP.4 (MI=51.1, δ=0.59)—were identified as potential improvements to the model. For indicative purposes, the CFA was performed for an adapted hierarchical model in which the weakest item (CA.2) was removed and the 2 error terms were added. The modified model performed slightly better than the unmodified models (Table 4).

### Convergent Validity

Following the UK study, we also explored the convergent validity of the original 20-item 4-factor model by correlating the observed mean factor scores with the mean scores for the 3 general normalization questions. Table 5 summarizes the findings. Weak correlations with the general normalization items were found for CO, CA, and RM (−.02≤*r*≤.27, 0.09≤*P*≤.81). The CP subscale had a moderate-to-strong correlation with the normalization items (.42≤*r*≤.59, *P*≤.05).

**Table 5:** Convergent validity: correlations of the total score and 4 factors with the general normalization items (Part B of the questionnaire).

General item	NPS^a^ (95% CI)	CO^b^ (95% CI)	CP^c^ (95% CI)	CA^d^ (95% CI)	RM^e^ (95% CI)
No. 1 Feels familiar	.26 (0.14 to 0.38)	.04^f^ (−0.09 to 0.17)	.50 (0.40 to 0.59)	.14 (0.01 to 0.26)	−.02^g^ (0.15 to 0.11)
No. 2 Is normal	.35 (0.23 to 0.46)	.13 (0.01 to −0.26)	.42 (0.31 to 0.52)	.27 (0.15 to 0.39)	.18 (0.05 to 0.30)
No. 3 Becomes normal	.32 (0.21 to 0.42)	.10^h^ (−0.03 to 0.22)	.59 (0.51 to 0.66)	.10^i^ (−0.02 to 0.22)	.03^j^ (−0.09 to 0.15)

^a^NPS: normalization process scale.

^b^CO: coherence.

^c^CP: cognitive participation.

^d^CA: collective action.

^e^RM: reflexive monitoring.

^f^*P*=.52.

^g^*P*=.81.

^h^*P*=.12.

^i^*P*=.09.

^j^*P*=.63; all other correlations are significant.

## Discussion

### Principal Findings

Accurate and reliable instruments for measuring implementation factors and progress are currently few but required to improve the uptake of eMH interventions in routine care [11,43,44]. For this study, we translated NoMAD from English to Dutch and sought to confirm its theorized 4-factor structure in mental health care settings.

Our suggests that the NoMAD can be used reliably in assessing normalization processes in Dutch mental care settings. Using structural equation modelling, the CFA showed that the 4-factor model fitted the observed data best. This finding points in the same direction as the English psychometric study (CFI=0.95, TLI=0.93, RMSEA=0.08, SRMR=0.03, estimator: maximum likelihood) [25]. The hierarchical model might prove useful in increasing the practical utility of the NoMAD questionnaire. It offers implementation practitioners and researchers with an overall normalization score enabling comparisons across implementation projects. In addition, the subscales scores can provide a more fine-grained understanding of normalization processes and aid in identifying specific areas for improvement.

Considering the factor loadings of both the 4-factor and hierarchical models and the measured internal consistency, improvement of the theorized model seems desirable. Most notably, the explained variance in item CA.2: [the intervention] disrupts working relationships, was below validation standards (see Figure 3). Even though the extent to which people are using eMH interventions in practice might influence their perceived disruptive nature, a possible explanation might be found in the limited variance in ratings for this item as 70% of the respondents strongly disagreed with this item. This might stem from differences in linguistic interpretation by the respondents. For example, it could be that the translation of the term disrupt in CA.2 has a more negative connotation among the Dutch respondents than it has among English native speakers, leading to a tendency toward negative responses in the Dutch sample. However, this is speculative, and we feel it is too early to discard the item. We suggest further deliberation on the theorized meaning of the latent and observed variables to determine the influence of sample characteristics, implementation objects, and linguistic differences in the item formulation before conclusive decisions about possible improvements to the theorized model can be made [26,33]. In addition, we observed an increase of responses rating items in the RM scale as not applicable. Given the novelty of eMH to the care setting, it might be that the respondents have had limited exposure to the intervention to reflect on its implementation. This corresponds with a sequential interpretation of the NPT constructs but is not the only approach to the ordering of NPT mechanisms [22], and we did not measure the stage or type of implementation trajectory that respondents were currently engaged with, making it too early to draw any conclusions on the item response patterns at this stage.

### Limitations

In view of the heterogeneity in implementation objects and health care settings included in current and previous NoMAD validation studies, the relevance of items should be taken in to account when administering the questionnaire to specific groups of respondents [24,25,45]. Although an open recruitment strategy was used for this study, it may be that the respondents had a natural inclination to partake in research or had a pioneering standpoint toward implementing eMH. This could have led to certain trends in the data that are not necessarily representative of the wider mental health care community involved in implementing eMH interventions. In this respect, it must be noted that sample groups 1 and 3 (mental health specialists and attendees of a national annual CBT congress, respectively) were convenience sampled. Sample 2 (48% of the pooled sample used in the CFA) was obtained through surveying members of a national professional association of general practice–based mental health nurse specialists [28]. We aimed to reduce selection bias by including these 3 sampling sources but results need to be interpreted with care because of the open design.

For the questionnaire, a 5-point Likert scale has been used for scoring the items. It goes beyond the scope of this study to discuss the consequences of this choice in depth, but it is important to note that we approached the individual responses as ordinal data because the numbers in Likert scales represent verbal statements and not numeric entities. The mean is commonly applied to summarize data allowing for calculating SDs and CIs. However, these indicators can be biased by outliers in non-normal distributions, possibly resulting in a distorted indication of the centrality of the data [46]. In addition, the approach to item nonresponse (or missingness) should be considered. Item nonresponse means that even though the respondent has participated in the study, data for certain items are unavailable for analysis [47]. In this study, respondents needed to either rate their agreement with the NoMAD items or indicate the item as not applicable.

For calculating a scale score, 2-rated items per scale were required. This approach might be limited in informing normalization processes as 50% (more for scales of more than 4 items) of the items in the same scale could be rated as not applicable. One possibility to reduce this nonignorable form of nonresponse is to apply a forced-choice approach by removing the option for respondents to rate an item as “not applicable” from the questionnaire. However, there is a risk that forcing a rating might lead to an artificial response when a respondent feels they do not have a choice. Another possibility is to apply theoretically defined and empirically confirmed cutoff for allowable missingness in calculating the scale scores.

A further methodological limitation of this study relates to the fit indices used to evaluate the models in the CFA. As the fit indices we used were developed for maximum likelihood–based parameter estimators, they should be interpreted with caution for ordinal data using robust Weighted Least Square estimators such as the WLSMV that we applied. It is argued that the distribution of the data and sample size have a consistent influence that might lead to overestimation of fit indices with ordinal data [48].

### Future Research

With this study, we have successfully contributed to the ambition of NoMAD in delivering a generic implementation measurement instrument for measuring normalization processes across different health care settings, including mental health [11,12,23-25,49,50]. Future research should assess relative predictive value and add to the practical interpretability and utility of the questionnaire. The hierarchical model provides the added value of a singular score for situations that require comparative evaluations of different implementation processes, while retaining the possibility to assess context-specific implementation processes at the construct level for understanding where implementation challenges exist in the development of effective and efficient implementation activities. However, and although interpretability of the sub-scale scores and the total NPS score does make sense from a mathematical perspective, the meaning and normativity of the scores in practice need to be established before these scores can serve implementation research and practice meaningfully. Future research should establish normative data and assess the implied factor structure of the hierarchical model in different datasets.

To increase comparability with the UK psychometric study, the 3 general normalization items were added to the questionnaire solely for assessing convergent validity [23-25]. Although this gives some indications of correlation of the NoMAD items with similar scales, the status of these 3 items is unclear. However, they do not constitute to the core of the questionnaire, and users are advised to disregard them. Instead, different measures of comparable constructs should be examined to establish a stronger assessment of convergent validity. Preferably, a multitrait-multimethod matrix should be used to strengthen conclusions about construct validity by using different methods such as organizational data on normalization success [34,51,52].

Test-retest reliability should be assessed to examine responsiveness of the questionnaire over time, to establish the ability of the questionnaire to measure changes when they occur. Responsiveness can be considered a measure of longitudinal validity and can be assessed by testing the predefined hypothesis about expected differences in changes between known samples at different time points [27]. As the duration to achieve implementation success can vary across context implementation object and implementation activities, careful consideration is needed regarding an appropriate time frame for repeat testing to assess responsiveness of the NoMAD questionnaire [53]. Applying a large-scale, stepped-wedge randomized controlled trial, NoMAD is used to measure change in normalization processes over time in the ImpleMentAll project (study protocol forthcoming) to test the effectiveness of tailored implementation compared with usual implementation activities for eMH interventions.

### Conclusions

Accurate and reliable assessment of implementation processes are needed to advance the implementation of eMH interventions in routine care. The translated NoMAD questionnaire proves to be a promising instrument in measuring implementation processes of innovative interventions in Dutch mental health care settings. The theorized 4-factor model approached the observed data acceptably, but there is room for improvement. The hierarchical model might prove useful in increasing the practical utility of the NoMAD questionnaire. Future research should add to the practical utility of the questionnaire by establishing normative data and assess the relative predictive value and responsiveness of the questionnaire over time.

## Appendix

Multimedia Appendix 1 Translated Normalization MeAsure Development-NL Questionnaire.

## Abbreviations

BC-MH: basic care-mental health
CA: collective action
CBT: cognitive behavioral therapy
CFA: confirmatory factor analysis
CFI: Comparative Fit Index
CO: coherence
CP: cognitive participation
eHealth: electronic health
eMH: eMental health
MI: modification index
NoMAD: Normalization MeAsure Development
NPS: normalization process scale
NPT: Normalization Process Theory
PC-MH: primary care-mental health
RE-AIM: Reach Effectiveness-Adoption Implementation Maintenance
RM: reflexive monitoring
RMSEA: Root Mean Square Error of Approximation
SC-MH: specialist care-mental health
SRMR: Standardized Root Mean Square Residual
TLI: Tucker Lewis Index
WLSMV: Weighted Least Square Means and Variances

## References

[1] Andersson G, Carlbring P, Lindefors N, Lindefors N, Andersson G (2016). History and Current Status of ICBT. Guided Internet-Based Treatments in Psychiatry.

[2] Titov N, Dear BF, Staples LG, Bennett-Levy J, Klein B, Rapee RM, Shann C, Richards D, Andersson G, Ritterband L, Purtell C, Bezuidenhout G, Johnston L, Nielssen OB (2015). MindSpot clinic: an accessible, efficient, and effective online treatment service for anxiety and depression. Psychiatr Serv.

[3] Mol M, Dozeman E, Provoost S, van Schaik A, Riper H, Smit JH (2018). Behind the scenes of online therapeutic feedback in blended therapy for depression: mixed-methods observational study. J Med Internet Res.

[4] Ruwaard JJ, Lange A, Schrieken B, Emmelkamp PM (2011). Efficacy and effectiveness of online cognitive behavioral treatment: a decade of interapy research. Stud Health Technol Inform.

[5] Hedman E, Ljótsson B, Rück C, Bergström J, Andersson G, Kaldo V, Jansson L, Andersson E, Andersson E, Blom K, El Alaoui S, Falk L, Ivarsson J, Nasri B, Rydh S, Lindefors N (2013). Effectiveness of internet-based cognitive behaviour therapy for panic disorder in routine psychiatric care. Acta Psychiatr Scand.

[6] Folker A, Mathiasen K, Lauridsen SM, Stenderup E, Dozeman E, Folker MP (2018). Implementing internet-delivered cognitive behavior therapy for common mental health disorders: A comparative case study of implementation challenges perceived by therapists and managers in five European internet services. Internet Interv.

[7] Mathiasen K, Riper H, Andersen TE, Roessler KK (2018). Guided internet-based cognitive behavioral therapy for adult depression and anxiety in routine secondary care: observational study. J Med Internet Res.

[8] Topooco N, Riper H, Araya R, Berking M, Brunn M, Chevreul K, Cieslak R, Ebert DD, Etchmendy E, Herrero R, Kleiboer A, Krieger T, García-Palacios A, Cerga-Pashoja A, Smoktunowicz E, Urech A, Vis C, Andersson G, E-COMPARED consortium (2017). Attitudes towards digital treatment for depression: a European stakeholder survey. Internet Interv.

[9] Titzler I, Saruhanjan K, Berking M, Riper H, Ebert DD (2018). Barriers and facilitators for the implementation of blended psychotherapy for depression: A qualitative pilot study of therapists' perspective. Internet Interv.

[10] Vis C, Mol M, Kleiboer A, Bührmann L, Finch T, Smit J, Riper H (2018). Improving implementation of eMental health for mood disorders in routine practice: systematic review of barriers and facilitating factors. JMIR Ment Health.

[11] Proctor EK, Silmere H, Raghavan R, Hovmand P, Aarons G, Bunger A, Griffey R, Hensley M (2011). Outcomes for implementation research: conceptual distinctions, measurement challenges, and research agenda. Adm Policy Ment Health.

[12] Lewis CC, Fischer S, Weiner BJ, Stanick C, Kim M, Martinez RG (2015). Outcomes for implementation science: an enhanced systematic review of instruments using evidence-based rating criteria. Implement Sci.

[13] Tabak RG, Khoong EC, Chambers DA, Brownson RC (2012). Bridging research and practice: models for dissemination and implementation research. Am J Prev Med.

[14] Nilsen P (2015). Making sense of implementation theories, models and frameworks. Implement Sci.

[15] Graham ID, Logan J, Harrison MB, Straus SE, Tetroe J, Caswell W, Robinson N (2006). Lost in knowledge translation: time for a map?. J Contin Educ Health Prof.

[16] Damschroder LJ, Aron DC, Keith RE, Kirsh SR, Alexander JA, Lowery JC (2009). Fostering implementation of health services research findings into practice: a consolidated framework for advancing implementation science. Implement Sci.

[17] Glasgow RE, Vogt TM, Boles SM (1999). Evaluating the public health impact of health promotion interventions: the RE-AIM framework. Am J Public Health.

[18] Ajzen I (1991). The theory of planned behavior. Organ Behav Hum Decis Process.

[19] May C, Finch TL (2009). Implementing, embedding, and integrating practices: an outline of normalization process theory. Sociology.

[20] May CR, Mair F, Finch T, MacFarlane A, Dowrick CF, Treweek S, Rapley T, Ballini L, Ong BN, Rogers A, Murray E, Elwyn G, Légaré F, Gunn J, Montori VM (2009). Development of a theory of implementation and integration: Normalization Process Theory. Implement Sci.

[21] Murray CE (2009). Diffusion of innovation theory: a bridge for the research–practice gap in counseling. J Couns Dev.

[22] May CR, Cummings A, Girling M, Bracher M, Mair FS, May C, Murray E, Myall M, Rapley T, Finch T (2018). Using normalization process theory in feasibility studies and process evaluations of complex healthcare interventions: a systematic review. Implement Sci.

[23] Finch TL, Rapley T, Girling M, Mair FS, Murray E, Treweek S, McColl E, Steen IN, May CR (2013). Improving the normalization of complex interventions: measure development based on normalization process theory (NoMAD): study protocol. Implement Sci.

[24] Rapley T, Girling M, Mair FS, Murray E, Treweek S, McColl E, Steen IN, May CR, Finch TL (2018). Improving the normalization of complex interventions: part 1-development of the NoMAD instrument for assessing implementation work based on normalization process theory (NPT). BMC Med Res Methodol.

[25] Finch T], Girling M, May CR, Mair FS, Murray E, Treweek S, McColl E, Steen IN, Cook C, Vernazza CR, Mackintosh N, Sharma S, Barbery G, Steele J, Rapley T (2018). Improving the normalization of complex interventions: part 2 - validation of the NoMAD instrument for assessing implementation work based on normalization process theory (NPT). BMC Med Res Methodol.

[26] Brown TA, Kenny DA (2006). Confirmatory factor analysis for applied research. Methodology in the Social Sciences (Series).

[27] Terwee CB, Bot SD, de Boer MiR, van der Windt DA, Knol DL, Dekker J, Bouter LM, de Vet HC (2007). Quality criteria were proposed for measurement properties of health status questionnaires. J Clin Epidemiol.

[28] Krijgsman J, Swinkels I, van Lettow B, de Jong J, Out K, Friele R, van Gennip L (2016). Meer dan Techniek: eHealth monitor 2016. eHealth monitor 2016.

[29] Brislin RW (1970). Back-translation for cross-cultural research. J Cross Cult Psychol.

[30] Willis G (2005). Cognitive Interviewing - A Tool for Improving Questionnaire Design.

[31] NetQ (2009). NetQL.

[32] Couper M, Tourangeau R, Conrad FG, Singer E (2016). Evaluating the effectiveness of visual analog scales. Social Science Computer Review.

[33] Hu LT, Bentler PM (1999). Cutoff criteria for fit indexes in covariance structure analysis: Conventional criteria versus new alternatives. Struct Equ Modeling.

[34] Hinkin TR (2016). A brief tutorial on the development of measures for use in survey questionnaires. Organ Res Methods.

[35] Field AZ, Miles J, Field Z (2012). Discovering Statistics Using R.

[36] RStudio Team (2016). RStudio.

[37] Revelle W (2018). The Comprehensive R Archive Network.

[38] Wickham H (2009). The Comprehensive R Archive Network.

[39] Decke D (2018). The Comprehensive R Archive Network.

[40] Rosseel Y (2012). The Comprehensive R Archive Network.

[41] Epskamp S (2017). The Comprehensive R Archive Network.

[42] Contributors (2018). The Comprehensive R Archive Network.

[43] Finch TL, Mair FS, O'Donnell C, Murray E, May CR (2012). From theory to 'measurement' in complex interventions: methodological lessons from the development of an e-health normalisation instrument. BMC Med Res Methodol.

[44] Vis C, Mol M, Kleiboer A, Bührmann Leah, Finch T, Smit J, Riper Heleen (2018). Improving Implementation of eMental Health for Mood Disorders in Routine Practice: Systematic Review of Barriers and Facilitating Factors. JMIR Ment Health.

[45] Jacobs SR, Weiner BJ, Bunger AC (2014). Context matters: measuring implementation climate among individuals and groups. Implement Sci.

[46] Jamieson S (2004). Likert scales: how to (ab)use them. Med Educ.

[47] Bernaards C, Sijtsma K (2000). Influence of Imputation and EM methods on factor analysis when item nonresponse in questionnaire data is nonignorable. Multivariate Behav Res.

[48] Nye C, Drasgow F (2010). Assessing goodness of fit: simple rules of thumb simply do not work. Organ Res Methods.

[49] Clinton-McHarg T, Yoong SL, Tzelepis F, Regan T, Fielding A, Skelton E, Kingsland M, Ooi JY, Wolfenden L (2016). Psychometric properties of implementation measures for public health and community settings and mapping of constructs against the Consolidated Framework for Implementation Research: a systematic review. Implement Sci.

[50] McEvoy R, Ballini L, Maltoni S, O'Donnell CA, Mair F, Macfarlane A (2014). A qualitative systematic review of studies using the normalization process theory to research implementation processes. Implement Sci.

[51] Campbell DT, Fiske DW (1959). Convergent and discriminant validation by the multitrait-multimethod matrix. Psychol Bull.

[52] Schmitt N, Stults DM (2016). Methodology review: analysis of multitrait-multimethod matrices. Appl Psychol Meas.

[53] Rogers E (2003). Diffusion of Innovations: Fifth Edition.

