# Supplementary material for: Toward an Objective Assessment of Implementation Processes for Innovations in Health Care: Psychometric Evaluation of the Normalization Measure Development (NoMAD) Questionnaire Among Mental Health Care Professionals
Source: J Med Internet Res. 2019 Feb 20;21(2):e12376. doi: 10.2196/12376 (PMC6401675; doi:10.2196/12376)
Supplement: Multimedia Appendix 1 [file jmir_v21i2e12376_app1.docx]

Multimedia Appendix 1: NoMAD-NL

| ***Samenhang*** | 1 = Helemaal mee eens. | 2 = Eens. | 3 = Eens noch oneens. | 4 = Oneens. | 5 = Helemaal mee oneens. | 0 = Niet van toepassing. |
| --- | --- | --- | --- | --- | --- | --- |
| CO.1. Ik overzie hoe de [interventie] zich onderscheiden van de gebruikelijke manier van werken. | O | O | O | O | O | O |
| CO.2. Collega’s in deze organisatie denken hetzelfde over het doel van de [interventie]. | O | O | O | O | O | O |
| CO.3. Ik begrijp hoe de [interventie] van invloed kan zijn op de aard van mijn eigen werk. | O | O | O | O | O | O |
| CO.4. Ik zie de potentiële meerwaarde van de [interventie] voor mijn werk. | O | O | O | O | O | O |
| ***Betrokkenheid*** | | | | | | |
| CP.1. Er zijn sleutelfiguren in de organisatie die de [interventie] kunnen doorvoeren en anderen betrekken. | O | O | O | O | O | O |
| CP.2. Ik geloof dat het toepassen van de [interventie] een legitiem onderdeel is van mijn rol in de organisatie. | O | O | O | O | O | O |
| CP.3. Ik sta er voor open om met collega’s op nieuwe manieren samen te werken bij het gebruik van de [interventie]. | O | O | O | O | O | O |
| CP.4. Ik blijf het gebruik van de [interventie] steunen. | O | O | O | O | O | O |
| ***Samen doen*** | | | | | | |
| CA.1. Ik kan de [interventie] gemakkelijk in mijn huidige werk integreren. | O | O | O | O | O | O |
| CA.2. De [interventie] ontregelt de werkverhoudingen binnen de organisatie. | O | O | O | O | O | O |
| CA.3. Ik heb vertrouwen in de vaardigheden van collega’s in het toepassen van de [interventie]. | O | O | O | O | O | O |
| CA.4. De [interventie] worden uitgevoerd door collega’s die daar vaardig in zijn. | O | O | O | O | O | O |
| CA.5. Er wordt voldoende training gegeven om collega’s in staat te stellen de [interventie] te implementeren. | O | O | O | O | O | O |
| CA.6. Er zijn voldoende middelen beschikbaar ter ondersteuning van de [interventie]. | O | O | O | O | O | O |
| CA.7. Het management ondersteunt de [interventie] adequaat. | O | O | O | O | O | O |
| ***Reflectie*** | | | | | | |
| RM.1. Ik ben op de hoogte van de (vak)literatuur over de effecten van de [interventie]. | O | O | O | O | O | O |
| RM.2. De collega’s zijn het erover eens dat de [interventie] de moeite waard zijn. | O | O | O | O | O | O |
| RM.3. Ik waardeer de effecten die de [interventie] op mijn werk hebben gehad. | O | O | O | O | O | O |
| RM.4. Feedback met betrekking tot de [interventie] kan worden gebruikt om deze in de toekomst te verbeteren. | O | O | O | O | O | O |
| RM.5. Ik kan aanpassen hoe ik met de [interventie] werk. | O | O | O | O | O | O |
